# Genome-Wide Association Mapping of Low-Nitrogen Tolerance in Hexaploid Oat Identifies a Suggestive Candidate Locus near *P5CDH* Associated with Root Dry Weight

**DOI:** 10.3390/genes17101314

**Published:** 2026-10-21

**Authors:** Yu Ling, Ziyang Wang, Shuhao Yu, Zihao Zhang, Haizhou Yang, Lihui Zhang

**Affiliations:** College of Grassland Science, Inner Mongolia Agricultural University, Hohhot 010011, Chinayanghaizhou@emails.imau.edu.cn (H.Y.);

**Keywords:** oat, low-nitrogen tolerance, low-nitrogen response, GWAS, genetic architecture, proline catabolism, *P5CDH*

## Abstract

Background/Objectives: Optimizing nitrogen fertilizer efficiency is crucial for sustainable agriculture, but the genetic basis of low-nitrogen (LN) tolerance in oat (*Avena sativa* L.), an important forage and grain crop, remains poorly characterized. Methods: This study investigated eleven LN tolerance phenotypes across 103 oat accessions using high-density resequencing markers, mixed-model association mapping (MLM), a FarmCPU sensitivity analysis, and Bayesian genetic architecture inference (BSLMM). Results: No locus achieved genome-wide Bonferroni significance, and Bayesian estimates indicated a low-to-moderate model-based PVE (0.10–0.23) with no clearly identified major-effect locus, suggesting that genomic selection represents a plausible future direction for LN tolerance in oat. At a suggestive threshold (*p* < 1 × 10^−5^), a cross-model intersection nominated a hypothesis-generating locus at 1D:349,799,042 associated with root dry weight. This locus showed haplotype-level phenotypic segregation (Wilcoxon *p* = 1.36 × 10^−5^) on the discovery panel and concordant signals between the two models (*r* = 0.92). The nearest nitrogen-metabolism-related gene, *P5CDH* (22.7 kb away), encodes Δ^1^-pyrroline-5-carboxylate dehydrogenase, which catalyses the terminal step of proline catabolism, and its wheat ortholog was significantly down-regulated under nitrogen deficiency. Conclusions: These findings provide a testable hypothesis linking proline catabolism to oat root plasticity under low nitrogen and a framework for validation in larger panels.

## 1. Introduction

Nitrogen (N) availability is a critical limiting factor for crop growth, development, and grain yield. However, excessive synthetic N fertilization has caused profound environmental damage, driving water eutrophication, soil acidification, and greenhouse gas emissions [1]. Breeding crop varieties with enhanced nitrogen use efficiency (NUE) provides a sustainable alternative to decrease chemical inputs while sustaining high yields [2,3]. Despite substantial progress in identifying the genetic components of NUE in major cereals—such as nitrate transport and signaling [4,5,6], amino acid metabolism, brassinosteroid signaling, and low-nitrogen tolerance regulators [7] in wheat and rice—the specific genetic basis of low-nitrogen (LN) tolerance in oat (*A. sativa*) remains poorly understood.

Plant responses to LN stress involve coordinated physiological and morphological shifts across shoot and root systems [8,9]. N-deficient plants typically suppress shoot biomass and photosynthesis while shifting metabolic resources toward root growth to maximize N acquisition [4]. This developmental plasticity is governed by high-affinity transporters, amino acid assimilation networks, and hormonal signaling cascades [4,10,11]. The genetic basis of this root plasticity—collectively termed root system architecture (RSA)—has been dissected through extensive root-QTL and genome-wide association studies in cereals [12,13,14], yet the root-trait QTLome remains sparsely characterized in hexaploid oat, particularly under nutrient stress. In forward-genetics mapping, standardizing phenotypic responses through relative performance metrics (the LN/NN ratio) effectively controls for confounding baseline differences in seedling vigor among diverse accessions [9,15,16]. This enables accurate quantification of genetic tolerance. Proline metabolism is central to the low-nitrogen response because proline serves a dual function as a compatible solute and a readily mobilized nitrogen reservoir [17,18]. Proline catabolism proceeds through a two-step mitochondrial pathway: proline dehydrogenase oxidizes proline to Δ^1^-pyrroline-5-carboxylate (P5C), which is subsequently oxidized to glutamate by Δ^1^-pyrroline-5-carboxylate dehydrogenase (*P5CDH*; *ALDH12*), the terminal and committed step of the pathway [19]. Because *P5CDH* activity is tightly coupled to nitrogen status, genes of the proline-catabolic pathway represent plausible regulatory nodes through which nitrogen availability shapes root growth and carbon–nitrogen partitioning [20,21].

Genome-wide association studies (GWAS) utilize historical recombination within diverse germplasm to resolve the genetic architecture of complex quantitative traits at high resolution [22]. However, executing GWAS in hexaploid crops introduces severe computational challenges due to large genome sizes and homoeologous gene blocks [23]. In addition, population structure can inflate false-positive rates, a problem that mixed linear models (MLMs) address by controlling for stratification and kinship, though often at the cost of false-negative inflation [24,25]. To optimize discovery power, multi-locus frameworks like FarmCPU utilize fixed-effect iterations with automated background-marker control to rescue major-effect associations [26]. In complex polyploids with limited panel sizes, coupling these multi-tiered statistical algorithms with robust phenotypic transformations is critical to ensure reliable computation.

In this study, we conducted a comprehensive genome-wide association and architecture analysis for eleven LN tolerance traits across a diverse panel of 103 oat accessions. By pairing a high-density genome-wide marker map with rank-based inverse normal transformed phenotypes, we leveraged single- and multi-locus frequentist models alongside Bayesian inference pipelines. Candidate genes within target windows were prioritized by integrating functional annotations, haplotype–phenotype association testing, and cross-species transcriptomic comparison. Our results nominate proline catabolism and carbon–nitrogen coordination as testable mechanistic hypotheses for oat root plasticity under nitrogen limitation.

## 2. Materials and Methods

### 2.1. Plant Materials and Phenotyping

This study evaluated a diverse panel of 103 oat accessions shared between a whole-genome resequencing cohort from our previous work (*n* = 189) [27] and a low-nitrogen phenotyping trial (*n* = 186). The phenotypic data were generated in the source study, a master’s thesis from the same institution [28] that is publicly available via the China National Knowledge Infrastructure (CNKI) and that included an association analysis of the full 186-accession panel based on the Oat 50K SNP array (Chengdu Tiancheng Weilai Technology Co., Ltd., Chengdu, China). The present analysis extends the source study by a ~10^3^-fold denser resequencing marker set, a multi-model analytical framework (MLM, a FarmCPU-based sensitivity analysis, and BSLMM), and cross-species candidate comparison, applied to the 103 accessions common to both datasets (a purely intersection-based criterion). Given the differences in marker density and panel composition, the lead loci identified here did not overlap those reported in the source study.

For phenotyping, seeds were surface-sterilized with 75% (*v*/*v*) ethanol (Sinopharm Chemical Reagent Co., Ltd., Shanghai, China) for 30 s, rinsed three times with sterile distilled water, and germinated in the dark (Appendix A.1). Seedlings were transferred to 96-cell hydroponic trays, each containing 900 mL of full-strength Hoagland solution [29] renewed every 3 d, and grown in a controlled-environment chamber at 20 °C day/16 °C night under a 16 h light/8 h dark photoperiod; light intensity, pH, aeration, and accession randomization were not recorded in the source study. Upon reaching the three-leaf-one-heart stage, plants were subjected to two nitrogen regimes for 15 d—normal nitrogen (NN; 2 mmol/L N) and low nitrogen (LN; 0.2 mmol/L N)—each with three biological replicates. Because standard Hoagland solution already contains nitrogen, a nitrogen-free Hoagland base was used during treatment, with nitrogen supplied solely by adding liquid ammonium nitrate (Sinopharm Chemical Reagent Co., Ltd., Shanghai, China) to the target concentrations (Appendix A.3). Each biological replicate consisted of one plant per accession; the three replicate plants (one per replicate) were harvested for biomass measurements, and accession means were calculated by averaging the three replicates. Thirteen agronomic traits spanning morphology, biomass allocation, and root system architecture were quantified and expressed as relative values (LN/NN ratios). To eliminate mathematical redundancy and avoid collinearity in the downstream GWAS models, two composite traits—total fresh weight (*TFW* = *SFW* + *RFW*) and total dry weight (*TDW* = *SDW* + *RDW*), where *SFW*, *RFW*, *SDW*, and *RDW* denote shoot fresh weight, root fresh weight, shoot dry weight, and root dry weight, respectively—were systematically excluded, retaining eleven traits for mapping. Detailed cultivation and phenotyping protocols, adhering to the procedures described by Chai [28], are provided in Appendix A.

### 2.2. Genotyping and Quality Control

Genotypic data (whole-genome resequencing SNPs) were obtained from a previous study by our group [27], in which 189 oat accessions were resequenced using 2 × 150 bp paired-end sequencing on the DNBSEQ-T7 platform (MGI Tech, Shenzhen, China) at a mean depth of 3.47× (average 46.13 G bases per sample). Raw reads were aligned to the oat reference genome (OT3098 v1) [30,31] using BWA-MEM with default parameters [32], and joint variant calling was performed with BCFtools (mpileup, v1.12) [33]. Variant-level filtering retained 64.5 million high-quality biallelic SNPs with QUAL > 30, read depth (DP) > 5, mapping quality (MQ) ≥ 40, Fisher strand bias (FS) ≤ 60, minor allele frequency (MAF) ≥ 0.03, and missing rate ≤ 30%.

Downstream quality control was implemented using PLINK2 (v2.0.0) [34,35] on the 103 overlapping phenotyped accessions. To accommodate the allo-hexaploid (AACCDD) genome of oat, each homeologous locus was treated as a diploid site following standard hexaploid crop GWAS protocols [22] and the mosaic structure of the OT3098 v1 reference assembly [30], with SNP effects modeled additively (0/1/2 allele dosage) without accounting for subgenome dosage. Furthermore, to prevent the spurious removal of homozygote-excess variants inherent to self-pollinating species, standard Hardy–Weinberg equilibrium (HWE) filtering was adjusted by applying the --keep-fewhet flag. This approach specifically retained biologically expected heterozygote deficits while eliminating heterozygote excesses indicative of genotyping errors or paralogous misalignments. The final filtering stringency retained biallelic SNPs with a minor allele frequency (MAF) ≥ 0.05, missingness ≤ 20%, and HWE --keep-fewhet (*p* < 1 × 10^−6^), yielding 48.4 million SNPs after excluding unplaced scaffolds. Of these, a further 5.6 million with 5–20% missingness were excluded by GEMMA’s default missingness filter (-miss 0.05), leaving 42.8 million variants for association testing.

### 2.3. Linkage Disequilibrium Pruning

Linkage disequilibrium (LD) pruning was performed using PLINK2 with a sliding window of 50 SNPs, a step size of 10 SNPs, and an *r*^2^ threshold of 0.2 (--indep-pairwise 50 10 0.2), which yielded 3.75 million approximately independent variants. Because the 50-SNP window does not correspond to a fixed physical distance, these pruned markers are not assumed to be fully independent, and some residual LD is expected; the parameters were chosen as a standard trade-off between marker independence and computational tractability. This pruned dataset was utilized exclusively for population structure characterization, including principal component analysis (PCA), ADMIXTURE, and kinship matrix estimation. Conversely, the full dataset of 48.4 million SNPs was retained for GWAS. Prioritizing the unpruned marker set for association mapping avoids the premature exclusion of potential causal variants in LD with other markers, which would otherwise weaken statistical signals and bias multiple-testing corrections.

### 2.4. Population Structure and Kinship Matrix Estimation

Principal component analysis (PCA) was performed on the LD-pruned marker set using PLINK2 to evaluate genetic variance coordinates. To further infer population stratification, ancestry coefficients were modeled using ADMIXTURE (v1.3.0) [36] across a range of ancestral clusters (K = 1 to 5), with the optimal cluster number determined based on the minimum cross-validation error threshold. Concurrently, a centered genomic relatedness matrix (kinship matrix) was calculated with GEMMA (v0.98.5) [37] using the -gk 1 option on the LD-pruned marker set (3,753,257 markers). To effectively control for confounding population stratification, the top principal components (PCs) were incorporated as fixed-effect covariates within the downstream genome-wide association models.

### 2.5. Association Mapping and Genetic Architecture Inference

#### 2.5.1. Phenotypic Data Transformation

Due to the inherent skewness typically observed in raw biomass trait ratios, normality was evaluated using the Shapiro–Wilk test. To ensure statistical validity for downstream genetic analyses, a rank-based inverse normal transformation (INT; Blom’s method) was systematically applied to all eleven traits. This transformation enforces an exact normal distribution while preserving the original rank order of the phenotypic values. All subsequent genome-wide association mappings were performed exclusively using these INT-transformed phenotypes.

#### 2.5.2. Mixed Linear Model Association Mapping

Primary association mapping was performed using the mixed linear model (MLM) framework implemented in GEMMA (v0.98.5), with marker significance evaluated via the Wald test (-lmm 4). The statistical model incorporated candidate SNP genotypes, an intercept, and the first three principal components (PCs) as fixed effects, while accounting for random polygenic effects through the genomic relatedness matrix. Two significance thresholds were established to control for multiple testing: a suggestive threshold of *p* < 1 × 10^−5^, and a stringent Bonferroni-corrected threshold of *p* < 1.17 × 10^−9^ (α = 0.05). Of the 48.4 million SNPs passing PLINK2 QC, variants failing the default quality-control filters of GEMMA were excluded prior to association testing, and the Bonferroni threshold was derived from the resulting 42,787,713 (≈42.8 million) analyzed variants. For comparison, a Bonferroni threshold based on the 3.75 million LD-pruned, approximately independent variants (*p* < 1.33 × 10^−8^) is also reported.

#### 2.5.3. Multi-Locus Analysis via FarmCPU

The FarmCPU algorithm was executed within the rMVP R package (v1.4.6) [38], initially intended as a complementary multi-locus mapping approach. Under the INT-transformed phenotypic matrix, however, no trait yielded adequate pseudo-QTNs to support iterative convergence: the model consistently terminated at loop 2 with seqQTN = NULL. In this degenerate configuration, FarmCPU functionally reduced to a PC-corrected general linear model without multi-locus background control. Its output was therefore retained solely as a sensitivity analysis of the MLM—with which it was highly concordant (Pearson *r* = 0.92 between −log_10_ *p* values across the 879 intersection SNPs)—and not as independent validation. Candidate loci were nevertheless defined as SNPs passing the suggestive threshold (*p* < 1 × 10^−5^) in both models; given the high correlation between the two *p*-value vectors, this intersection should be interpreted as a consistency filter rather than as orthogonal confirmation.

#### 2.5.4. Genetic Architecture Inference via BSLMM

To characterize the overall genetic architecture of the target traits, the Bayesian sparse linear mixed model (BSLMM) [39] was fitted within GEMMA using the -bslmm 1 option. The Markov chain Monte Carlo (MCMC) algorithm was executed with 5000 burn-in iterations (-w 5000) followed by 50,000 sampling steps (-s 50,000), of which every 10th sample was recorded (5000 retained samples); post-burn-in trace inspection indicated stable sampling. This framework estimated the proportion of phenotypic variance explained by all genotyped SNPs for the inverse-normal-transformed phenotypes (PVE) and the proportion of PVE attributable to sparse, large-effect loci *ρ*. Here, PVE represents the model-based narrow-sense heritability (*h*^2^) for the inverse-normal-transformed phenotypes, rather than a broad-sense heritability estimate. The BSLMM was fitted using the full set of 48,400,552 SNPs, with the kinship matrix (computed from the LD-pruned 3,753,257 markers) as the random-effect covariance, as the Bayesian framework inherently accounts for background population stratification; this kinship matrix was the same as that used in the MLM. Hyperparameter estimates for PVE and *ρ* were derived by calculating the posterior means from the “pve” and “rho” outputs within the hyp.txt file across the post-burn-in sampling distribution. These metrics provided an evaluation of genetic architecture independent of strict significance-based locus discovery thresholds.

### 2.6. Candidate Gene Identification and Functional Annotation

Candidate loci were delineated as SNPs simultaneously passing the suggestive threshold (*p* < 1 × 10^−5^) in both the MLM and FarmCPU frameworks. Candidate genes were mined within a ±50 kb flanking window centered on each lead SNP. Because oat exhibits extensive linkage disequilibrium (*r*^2^ > 0.4 extending beyond 1 Mb) [27], this window—narrower than the LD extent—was used to keep the number of candidate genes tractable. To address the extensive transcript redundancy arising from multiple transcript models within the OT3098 v1 annotation, gene models with overlapping coordinates were collapsed into unique genomic loci prior to downstream interrogation. To identify the most promising targets for experimental validation, candidate loci were systematically prioritized based on three criteria: (1) detection at the suggestive threshold in both association models with concordant effect directions, (2) location within ±50 kb of a functionally annotated gene with established relevance to nitrogen metabolism, which served as the primary discriminator among suggestive loci rather than absolute peak significance; and (3) confirmation of genotype-phenotype segregation by haplotype-phenotype association testing within the discovery panel, which constitutes a sensitivity analysis rather than an independent validation (Section 2.9).

### 2.7. Functional Annotation and Enrichment Analysis

Functional annotation was executed via eggNOG6-mapper (v2.1.15) [40] against the eggNOG6 5.0 database, using gene models from the OT3098 v1 annotation. GO and KEGG [41] enrichment was performed with clusterProfiler (v4.18.4, hypergeometric test) [42] using Benjamini–Hochberg correction (*q* < 0.05), testing candidate genes against a background of 15,916 GO- and 17,631 KEGG-annotated genes (per-trait gene counts in Table S8). Enriched GO terms were evaluated across biological process, cellular component, and molecular function. To avoid annotation artifacts, redundant gene models with overlapping coordinates were consolidated, and animal- and microbe-specific GO/KEGG terms were removed.

### 2.8. Cross-Species Comparative Genomics and Transcriptomic Support

To identify cross-species orthologs, the representative protein sequence of each oat candidate gene was projected against the wheat IWGSC v2.1 proteome [43] via BLASTp (v2.10.1+) [44]. Homology searches were restricted to the top reciprocal best hit per gene, subject to strict filtration thresholds of an E-value < 1 × 10^−5^ and a sequence identity > 70%. The identified wheat orthologs were subsequently cross-referenced against multiple independent, peer-reviewed wheat low-nitrogen transcriptome datasets [45,46,47]. These datasets comprised: (i) leaf and root transcriptomes of the nitrogen-sensitive wheat cultivar Shannong 29 grown hydroponically under complete (5 mmol L^−1^ NH_4_NO_3_) versus nitrogen-free (0 mmol L^−1^) nutrient solution and sampled 3 days after transplanting, in three biological replicates, yielding 3949 leaf and 3911 root DEGs (*p* < 0.05, |log_2_FC| ≥ 1, DESeq2) [45]; (ii) flag-leaf transcriptomes of two wheat near-isogenic lines contrasting for nitrogen-use efficiency (1Y, high-NUE; 1W, low-NUE) grown in a pot experiment under 0 versus 300 kg ha^−1^ nitrogen and sampled at the anthesis stage, in three biological replicates (12 libraries), with DEGs defined by adjusted *p* < 0.05 and |log_2_FC| > 1 (DESeq2, v1.16.1) [46]; and (iii) root and shoot transcriptomes of two bread-wheat cultivars (PBW677, N-efficient; PBW703, N-inefficient) grown under nitrogen-control (120 kg ha^−1^ urea) versus nitrogen-stress conditions and sampled 24 h after urea application, in three biological replicates, yielding 2406 DEGs (*p* < 0.05, |log_2_FC| > 2, Cuffdiff, v2.2.1) [47]. In all cases, the published DEG lists were used directly; no re-analysis of the raw data was performed. Oat candidate loci were prioritized as cross-species supported candidates if their corresponding wheat orthologs exhibited significant differential expression under nitrogen-limitation regimes in at least one independent dataset.

### 2.9. Haplotype–Phenotype Association Analysis

Haplotype–phenotype associations for high-confidence loci were evaluated by stratifying the oat panel into candidate haplotype groups according to their alleles at the primary index SNP. Inter-group phenotypic variances were dynamically resolved using a two-sided Wilcoxon rank-sum test. Furthermore, to preclude potential false-positive associations stemming from hidden stratification, the distribution of the first principal component (PC1) was cross-checked between genotype groups to ensure population-structure alignment. Because this comparison uses the same accessions and phenotypes as the discovery scan, it constitutes a sensitivity analysis of the association rather than an independent validation.

## 3. Results

### 3.1. Phenotypic Variation and Normality Evaluation

The evaluated panel of 103 oat accessions displayed extensive phenotypic variation across all eleven low-nitrogen (LN) tolerance traits (Table 1 and Table S1; Figure S1). Phenotypic variability was heavily accentuated in biomass accumulation metrics, with root dry weight (RDW) showing the widest dispersion (CV = 67.4%, mean = 1.32, range: 0.12–5.48), followed closely by shoot dry weight (SDW; CV = 65.1%) and root fresh weight (RFW; CV = 49.7%). In contrast, morphological and root architecture traits remained comparatively stable, maintaining narrower distribution coefficients (CV: 10.7–29.5%). The raw distributions of biomass-related traits (SFW, RFW, SDW, and RDW) were severely right-skewed, characterized by a substantial proportion of accessions achieving LN/NN ratios exceeding 2.0 (Figure S1), which significantly deviated from a Gaussian distribution (Shapiro–Wilk test, *p* < 1 × 10^−8^; Table 1). Implementation of the rank-based inverse normal transformation (INT) successfully corrected these distributional distortions, enforcing uniform normality across all eleven traits (Shapiro–Wilk test, *p* ≈ 1.0). Consequently, these INT-transformed phenotypes were utilized as the baseline matrix for all subsequent downstream association mapping.

### 3.2. Population Structure and Kinship Assessment

**Figure 1:**
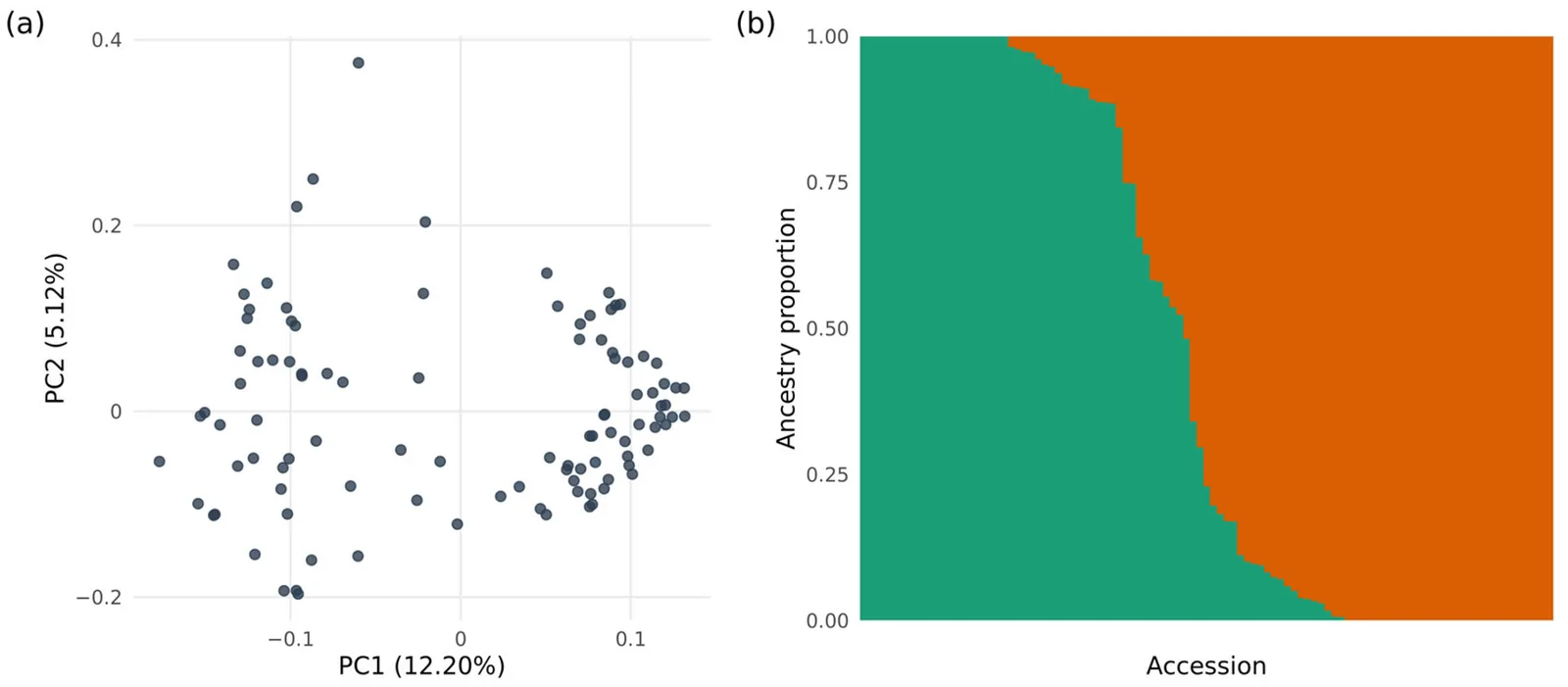
Genomic stratification and ancestral composition of the 103 oat accessions. (**a**) Principal component analysis (PCA) profiling based on the first two major axes, with parentheses indicating the percentage of genetic variance explained by PC1 (12.20%) and PC2 (5.12%). (**b**) Model-based ancestry proportions inferred via ADMIXTURE at the optimal clustering configuration (*K* = 2); each vertical column indexes a distinct germplasm accession, with the structural coloring reflecting the relative assignment to the two inferred ancestral gene pools.

The 103 oat accessions exhibited mild-to-moderate population structure, where PC1, PC2, and PC3 captured 12.20%, 5.12%, and 4.76% of the genetic variance, respectively (Figure 1a). This stratification was corroborated by ADMIXTURE modeling, which identified *K* = 2 as the optimal subpopulation architecture based on the minimum cross-validation error (Figure 1b). The two inferred ancestral clusters closely aligned with the spatial separation observed along the PC1 trajectory. Pairwise kinship coefficients revealed a continuous genetic relatedness profile across the panel, reflecting balanced relatedness parameters suitable for controlled genome-wide association testing.

### 3.3. Genome-Wide Association Mapping and Genetic Architecture Profiles

**Figure 2:**
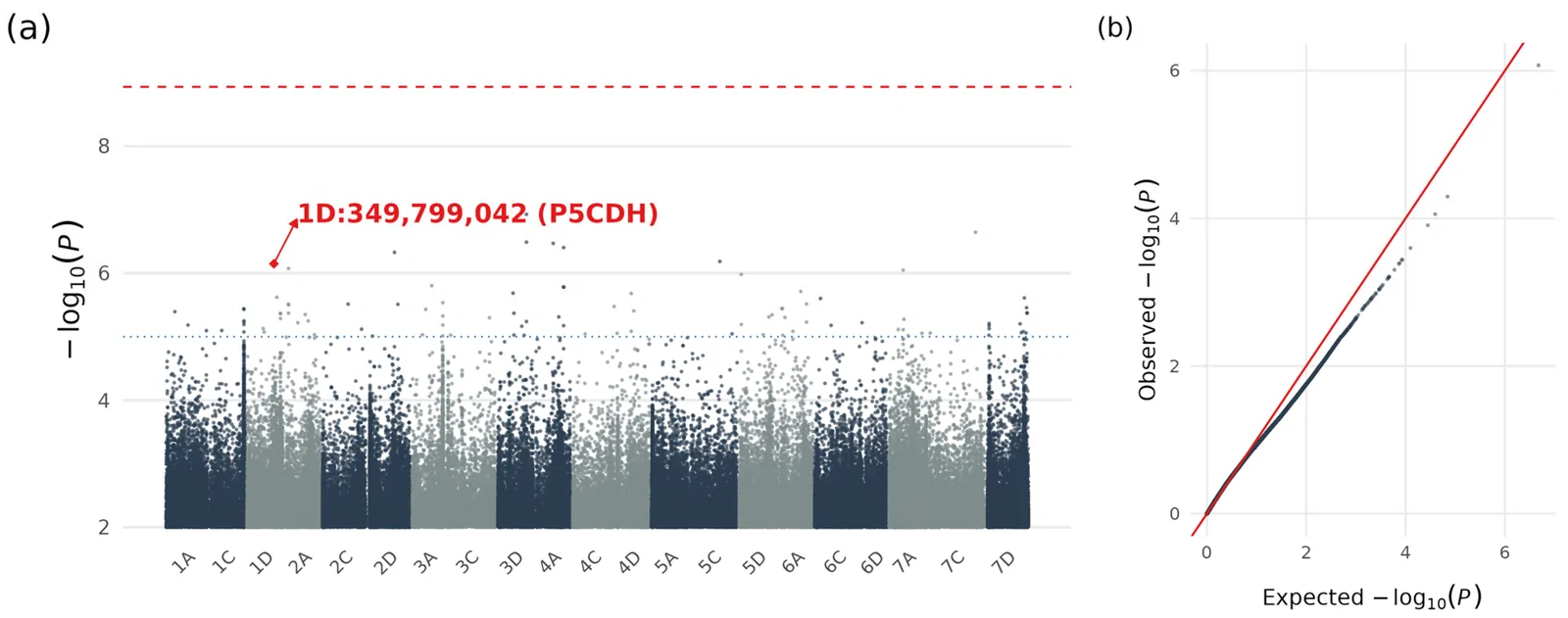
Genome-wide association mapping for root dry weight (RDW). (**a**) Genome-wide Manhattan plot derived from the mixed linear model (MLM) profiling applied to rank-based inverse normal transformed phenotypes. The horizontal dashed lines demarcate the suggestive threshold (*p* = 1 × 10^−5^, in blue) and the stringent Bonferroni-corrected threshold (*p* = 1.17 × 10^−9^, in red), with the prioritized target candidate locus 1D:349,799,042 explicitly indexed and highlighted. (**b**) Quantile–quantile (Q–Q) plot contrasting observed versus expected −log_10_(*p*) statistics. The calculated genomic inflation factor (*λ* = 1.09) demonstrates adequate mitigation of confounding population stratification artifacts across the mapping framework.

Association mapping via the MLM identified suggestive single-nucleotide polymorphisms (*p* < 1 × 10^−5^) across all eleven evaluated traits, with the number of suggestive markers ranging from 43 to 288 per trait, representing 41 to 138 independent genomic regions after LD-based clumping (*r*^2^ > 0.2, ±1 Mb) (Figure 2, Figure S2 and Figure S3, and Table 2 and Table S2). Notably, no marker surpassed the conservative genome-wide Bonferroni-corrected significance threshold (*p* < 1.17 × 10^−9^) for any investigated trait (Table 2). The suggestive pool is subject to substantial multiplicity: under the complete null and assuming test independence, 42.8 million tests at *p* < 1 × 10^−5^ would be expected to yield approximately 428 false positives per trait, whereas extensive linkage disequilibrium reduces the effective number of independent tests—approximately 3.75 million after pruning, implying roughly 37 expected hits—so that the true null expectation lies between these two bounds. The observed counts (43–288) fall within this bracket and therefore cannot be distinguished from the null expectation without empirical calibration. All candidate loci derived from the suggestive pool are consequently interpreted strictly as hypothesis-generating. The FarmCPU sensitivity model yielded 49 to 315 suggestive SNPs per trait with zero Bonferroni-significant detections and allelic effect directions consistent with the MLM. Genomic control indicated moderate inflation (λ = 1.09 for RDW), consistent with residual polygenic signal rather than uncontrolled stratification.

Concurrently, Bayesian inference via BSLMM partitioned the trait variation, estimating low-to-moderate PVE across the panel (posterior mean = 0.102–0.229; Table 2). The proportion of PVE driven by sparse major-effect loci (*ρ*) could not be reliably estimated, with 95% credible intervals spanning nearly the full [0, 1] range (Table 2). For the key target trait RDW, the estimated number of sparse-effect variants (*n_γ_*) was similarly uncertain (posterior mean ≈ 19, 95% CI [1, 46]).

### 3.4. Prioritization and Multidimensional Characterization of the Candidate Locus 1D:349,799,042

Systematic screening of the overlapping suggestive loci (*p* < 1 × 10^−5^) prioritized a single locus at 1D:349,799,042 associated with root dry weight (RDW), based on the presence of a functionally annotated, directly nitrogen-metabolism-related gene within a ±50 kb flanking window. Notably, lead SNPs for the other evaluated traits primarily resided in regions lacking nitrogen-metabolism-related gene annotations (Table S3). Although the strongest RDW signal (3D:372,973,527, *p* = 1.18 × 10^−7^) was also detected by both MLM and FarmCPU, it was deprioritized solely because its flanking genes (copine, helicase) lack nitrogen-metabolism annotations. Consequently, the 1D locus (*p* = 7.10 × 10^−7^) was prioritized based on its direct metabolic relevance rather than absolute statistical peak magnitude. Although this locus did not reach genome-wide significance, the index variant explained approximately 23% of the inverse-normal-transformed RDW variance (*R*^2^ = 0.23).

**Figure 3:**
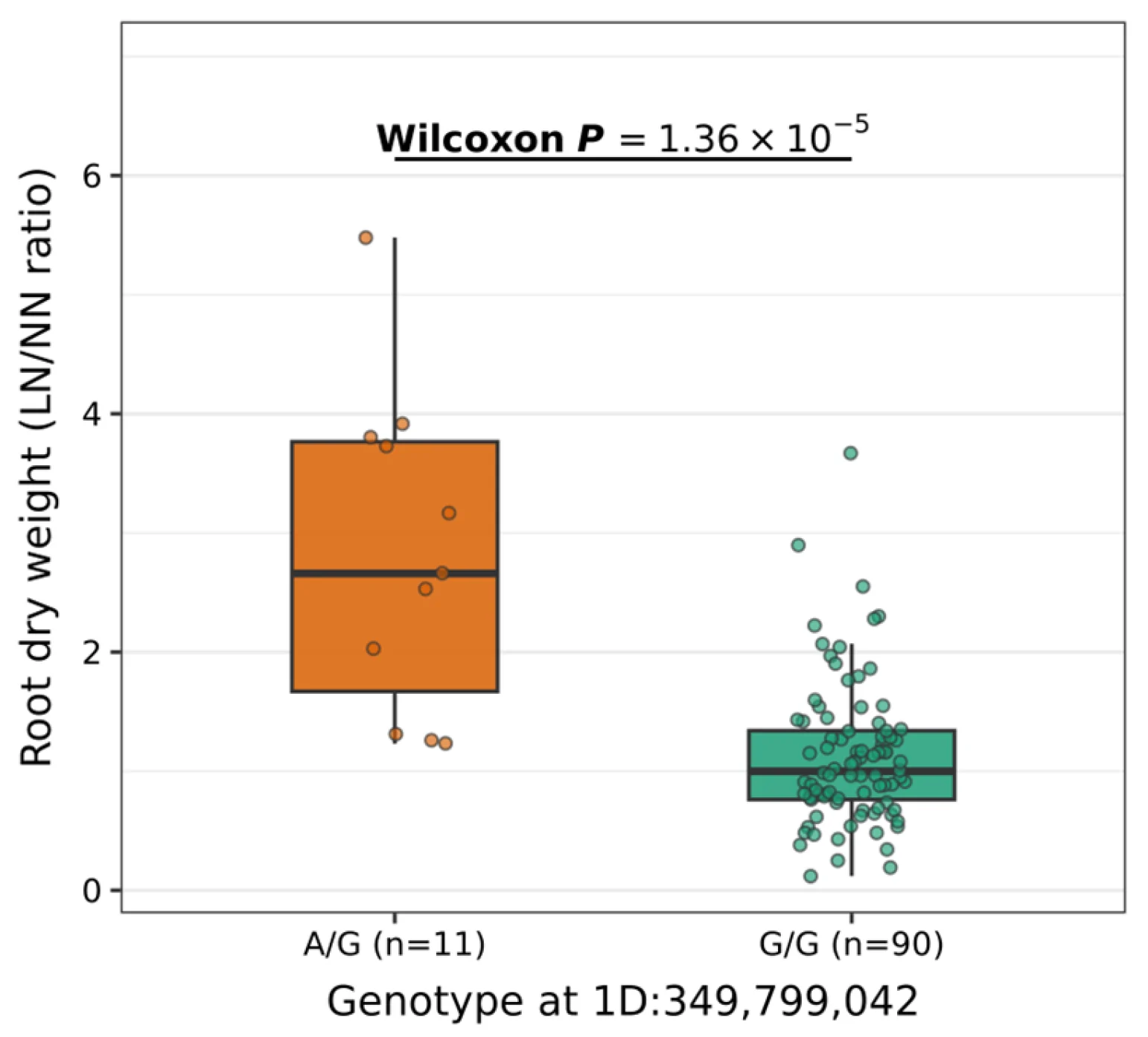
Haplotype–phenotype segregation at the prioritized candidate locus 1D:349,799,042. Boxplot illustrates the root dry weight (RDW, expressed as the low-nitrogen to normal-nitrogen [LN/NN] ratio) of oat accessions harboring the heterozygous A/G genotype (*n* = 11) versus the alternative homozygous G/G genotype (*n* = 90). Center lines represent the median, box boundaries span the interquartile range (IQR), and whiskers extend to the most extreme data points within 1.5 × IQR; individual accessions are superimposed as jittered markers. Statistical significance was evaluated via a two-sided Wilcoxon rank-sum test (*p* = 1.36 × 10^−5^).

This prioritized locus was supported by several lines of evidence, which are correlated rather than independent. Cross-model alignment was indicated by congruent detection between the MLM (*p* = 7.10 × 10^−7^) and FarmCPU (*p* = 1.02 × 10^−6^) algorithms, with concordant effect directions between the MLM and FarmCPU for every trait, although FarmCPU reduced to a general linear model for several traits (Section 2.5.3), so this is not independent replication. Concurrently, this locus exhibited consistent positive effect directions across most traits—including RDW, RFW, SDW, and TRL (Table 3), with the notable exception of LW (*β* = −0.531). Among the eleven traits, significant effects (95% CI excluding zero) were confined to the biomass traits RDW, RFW, and SDW, whereas morphological and chlorophyll traits showed no effect. However, only RDW reached the suggestive threshold, and because these traits are correlated, such consistency is expected and does not by itself imply pleiotropy. Haplotype–phenotype association testing revealed that accessions carrying the heterozygous A/G genotype (*n* = 11) manifested substantially elevated RDW ratios compared to G/G homozygotes (*n* = 90), with a mean LN/NN ratio of 2.83 versus 1.12 (Wilcoxon test, *p* = 1.36 × 10^−5^; Figure 3). At this site, no A/A homozygote was observed, and two accessions lacked genotype calls (11 A/G + 90 G/G + 2 missing = 103). The association therefore reflects a carrier contrast, from which allelic dosage cannot be distinguished; its nominal significance should be interpreted in the discovery-sample setting. Because the resequencing depth was low (3.47×), heterozygous calls at low-frequency variants are uncertain, and this genotype pattern could reflect either a genuine rare variant or a genotyping artifact; accordingly, we treat this variant as a candidate requiring independent confirmation. No significant PC1 difference was observed between genotype groups (*p* = 0.619), although the low carrier count (*n* = 11) limits the power of this check. Due to the low-frequency nature of this index SNP (MAF = 5.4%), standard LD parameters (*r*^2^ and *D*′) were statistically constrained; hence, further fine-mapping was bypassed in favor of a functionally driven prioritization strategy.

**Figure 4:**
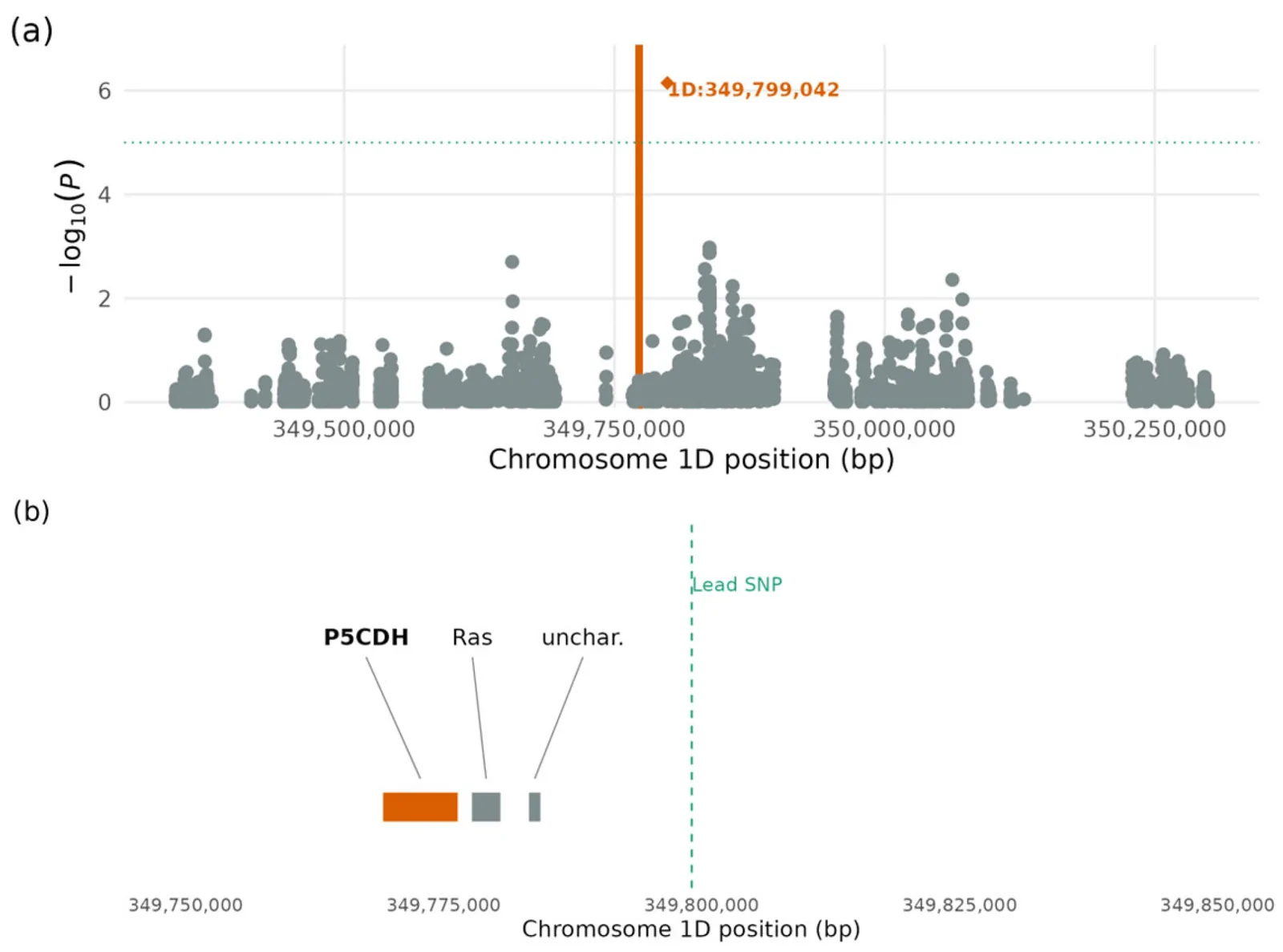
Local Manhattan profile and genomic architecture of the prioritized candidate locus on chromosome 1D. (**a**) Regional Manhattan plot displaying −log_10_(*p*) values within a ±500 kb window surrounding the lead index marker 1D:349,799,042 (highlighted as an orange diamond); the orange-shaded rectangle delineates the genomic physical boundaries of the *P5CDH* gene model. The horizontal dashed line marks the suggestive significance threshold (*p* = 1 × 10^−5^). (**b**) Schematic illustration of gene annotations spanning the immediate ± 50 kb genomic neighborhood, with the targeted *P5CDH* candidate gene structurally detailed and highlighted in orange.

The ±50 kb genomic neighborhood flanking 1D:349,799,042 contained a single functionally relevant gene, *P5CDH* (*Pepsico2_Contig19990*; coordinates 1D:349,769,137–349,776,370), located 22.7 kb from the index SNP and encoding Δ^1^-pyrroline-5-carboxylate dehydrogenase (*ALDH12*; KEGG K00294), which catalyzes the final step of proline catabolism (Figure 4; Table S4). The remaining gene models in this block (a Ras-family member and two uncharacterized transcripts) lacked apparent nitrogen-related descriptions. Comparative genomics mapped *OatP5CDH* to its wheat ortholog *TraesCS1A02G357900* (93.5% sequence identity, E-value = 0). Integration with external transcriptomic matrices confirmed that this wheat counterpart was significantly down-regulated under nitrogen deficiency in both evaluated near-isogenic lines (1Y: log_2_FC = −1.42, adjusted *p* = 2.98 × 10^−16^; 1W: log_2_FC= −1.45, adjusted *p* = 7.40 × 10^−18^) [46].

### 3.5. GO/KEGG Enrichment Analysis and Metabolic Adaptation

**Figure 5:**
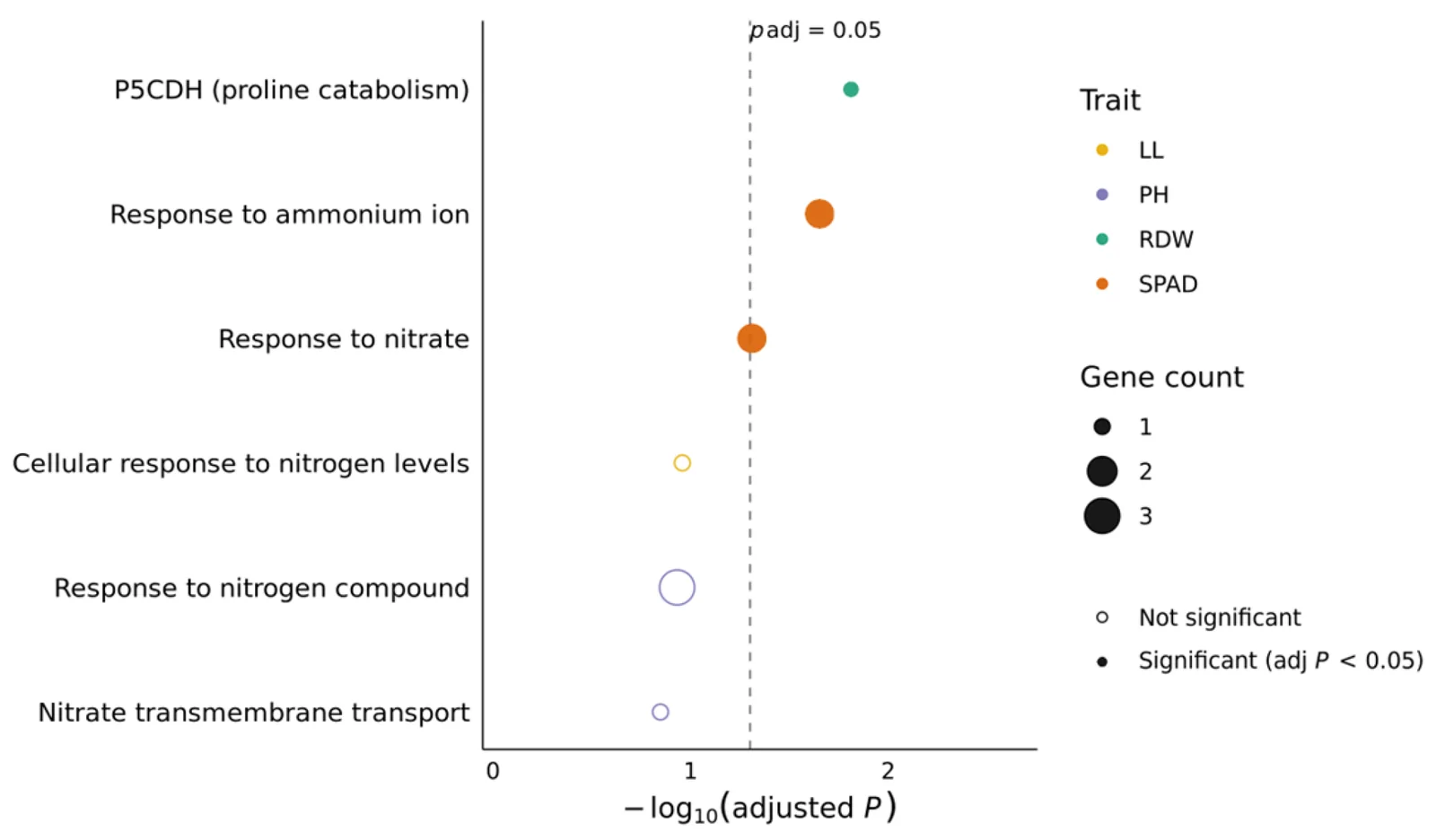
Functional enrichment map of nitrogen-responsive and proline-metabolic pathways among candidate genes across different traits. GO terms and KEGG pathways were analyzed based on the candidate gene sets for each phenotype, with the dashed baseline indicating the significance threshold (adjusted *p* = 0.05). Candidate genes associated with the SPAD trait were significantly enriched in nitrogen-responsive pathways, particularly involving *phosphoenolpyruvate carboxylase* (*PEPC*). In addition, the RDW candidate set contained the *P5CDH* (proline catabolism) entry, because this gene was the basis on which the locus was prioritized in Section 3.4. In contrast, nitrogen-transport or metabolic annotations related to plant height (PH) and leaf length (LL) were not significantly enriched (adjusted *p* ≥ 0.05) after removing potential metazoan-derived annotation artifacts.

Functional enrichment of the 1025 collapsed candidate genes shifted toward broader nitrogen-responsive signaling networks rather than classical uptake or assimilation cascades (Figure 5; Table S5), with core functional families (e.g., NRT transporters, GS/GOGAT) completely absent from enriched pools. Conversely, SPAD-associated candidates were significantly enriched for “response to ammonium ion” (adjusted *p* = 0.022) and “response to nitrate” (adjusted *p* = 0.049). This response was steered by phosphoenolpyruvate carboxylase (PEPC), a conserved carbon-metabolism enzyme in the Poaceae [48]. This metabolic alignment was further supported by the significant down-regulation of orthologous wheat PEPC models under nitrogen starvation (log_2_FC= −1.3 to −2.6, Table S6) [45], reflecting fine-tuned carbon–nitrogen coordination under LN stress.

For the RDW trait, an apparent enrichment of “heparan sulfate proteoglycan biosynthesis” was flagged as an in silico annotation-transfer artifact—where animal-derived terms were misassigned to plant orthologs—and was subsequently removed. Ultimately, the curated RDW KEGG network successfully captured the unique low-nitrogen-relevant 1-pyrroline-5-carboxylate dehydrogenase (K00294; *P5CDH*) entry.

## 4. Discussion

### 4.1. Model-Based PVE and Analytical Justifications

The absence of genome-wide Bonferroni-significant associations under robust frequentist frameworks points to the intricate genetic architecture underlying oat LN tolerance traits. Given that genomic inflation tracking ruled out background stratification artifacts, the low-to-moderate PVE (0.102–0.229) and sparse-effect proportions (*ρ* = 0.438–0.594) resolved by BSLMM could not distinguish the sparse and polygenic components, with 95% credible intervals for ρ spanning nearly [0, 1], largely owing to the limited statistical power of our small sample (*n* = 103) and the uncertainty inherent to Bayesian posterior estimation.

In small-to-moderate mapping populations, classical multi-testing adjustments frequently obscure genuine causal loci with subtle to intermediate effect sizes. Consequently, the absence of stringent peaks stems from restricted statistical power rather than a deficiency in actionable genetic variance. While the high SNP density improves fine-mapping resolution for loci that are detected, it does not compensate for the reduced power arising from the small sample (*n* = 103) or from extensive LD. This methodological boundary heavily justifies the implementation of a dual-model intersection strategy (MLM ∩ FarmCPU) at a relaxed suggestive threshold (*p* < 1 × 10^−5^). By requiring strict cross-model convergence under less conservative stringency, this pipeline bypassed the polygenic resolution bottleneck, enabling the identification of candidate loci tailored for downstream functional validation.

### 4.2. Proline Catabolism and the Candidate Gene P5CDH

The candidate locus 1D:349,799,042 (RDW) is supported by cross-model concordance and haplotype-level phenotypic segregation. This locus lies 22.7 kb from *P5CDH* (*Pepsico2_Contig19990*), a well-conserved single-copy gene encoding Δ^1^-pyrroline-5-carboxylate dehydrogenase to catalyze the final step of proline breakdown into glutamate [20,49]. This functional hypothesis is reinforced by the broader SPAD and RDW enrichment profiles, which pinpointed a tight carbon–nitrogen metabolic alignment rather than isolated nutrient-assimilation pathways.

Proline plays a crucial dual role during nitrogen starvation, operating as an osmoregulatory solute and a nitrogen reservoir for vegetative remobilization [17,18]. The transcriptional repression of *P5CDH* under nitrogen deficiency [46] would block the terminal step of proline catabolism and is therefore consistent with a strategy of proline accumulation as an osmolyte and nitrogen reservoir [17,18,50]. Active proline catabolic flux has, however, also been implicated in root development, where it fine-tunes ROS gradients and maintains root apical meristem activity [21]. These two models pull in opposite directions, and the link between *P5CDH* repression and root growth cannot be resolved from the present data; doing so will require direct measurement of oat *P5CDH* expression and free-proline pools under NN and LN in accessions carrying the A/G and G/G haplotypes. The association pattern at the index SNP—an allele contrast in root dry-matter accumulation—is compatible with a role for proline metabolism in the low-nitrogen response, and the recruitment of proline metabolism under nutrient stress mirrors conserved responses in other crop species under drought and salinity, suggesting that the oat *P5CDH* response forms part of a generalized stress-tolerance program.

Although these multi-dimensional datasets are highly consistent, they represent a correlation rather than proof of causality. Due to the minor allele frequency limits of the index SNP (MAF = 5.4%), linkage disequilibrium-based fine-mapping was bypassed in favor of functional prioritization within the ±50 kb window. Future functional genomics studies in oat are therefore essential to definitively validate the regulatory role of *P5CDH*.

### 4.3. Evolutionary Conservation of Cereal Proline Catabolism

The *P5CDH*-associated proline response is unlikely to be an oat-specific phenomenon. *P5CDH* is tightly conserved as a single-copy gene across the grass lineage—including hexaploid and durum wheat, Aegilops tauschii, barley, maize, and rice—featuring a preserved exonic structure and a conserved 22 bp proline-response element immediately upstream of the coding region [49]. Here, “single-copy” refers to a single locus rather than triplicated homeologs, as established by Ayliffe et al. [49], who found *P5CDH* to be single-copy even in hexaploid wheat; this status was not re-assessed against the oat A/C/D subgenomes in the present study.

In the literature, the regulatory direction of *P5CDH* is strikingly context-dependent rather than entirely uniform. For instance, under drought and hyperosmotic stresses, rice *OsP5CDH* is transcriptionally induced, not repressed [19], a mechanism thought to prevent excessive P5C–proline cycling and the associated reactive oxygen species (ROS) production [51]. Interestingly, while maize (*ZmALDH12*) and *Arabidopsis* (*AtALDH12A1*) *P5CDH* variants respond strongly to desiccation and senescence rather than nutrient availability, direct low-nitrogen-induced transcriptional down-regulation has so far been confined to wheat orthologs [46].

Therefore, the low-nitrogen-induced suppression observed for the wheat ortholog—and corroborated by our mapping insights in oat—is best interpreted as the recruitment of an early, acute-stress mechanism that initiates proline pooling rather than a nitrogen-exclusive pathway. This evolutionary conservation underscores that the low-nitrogen response of oat *P5CDH* is integrated into a broader, cross-species stress-adaptation program, strongly supporting its plausibility as a genuine contributor to oat root plasticity rather than an isolated, trait-specific statistical artifact.

### 4.4. Implications for Oat Breeding Strategies

The structural profile of marker-trait associations resolved under our present experimental framework carries practical implications for tailoring subsequent low-nitrogen tolerance (LN) breeding programs. Because no singular genomic hotspot transcended strict genome-wide Bonferroni significance, traditional marker-assisted selection (MAS)—which relies heavily on elite major-effect QTLs—is unlikely to yield effective genetic gain within this breeding panel. If the genetic architecture of oat LN tolerance is indeed governed by a polygenic background without a confidently detected major-effect locus, as our collective dataset suggests, deploying genomic selection (GS) represents a plausible future direction, a case made empirically for oat by Asoro et al. [52]. Unlike the single-marker approach, GS models leverage whole-genome marker densities to capture the cumulative effects of non-zero genetic variance, while avoiding reliance on individually significant markers. However, the present study does not directly compare GS with MAS or evaluate prediction accuracy, so this inference remains provisional. Nevertheless, formulating definitive long-term breeding roadmaps remains contingent upon expanding the mapping cohort size, a critical prerequisite to fully distinguish a highly dispersed polygenic landscape from statistical power limitations. More broadly, root system architecture is an established breeding target in cereals [12,14], and the root-trait loci reported here extend this QTLome for oat, supporting marker-informed selection for root architecture.

### 4.5. Study Limitations and Future Perspectives

Several limitations in the current study define clear trajectories for future research. First, fine-mapping within the target locus was restricted because the index marker is a low-frequency variant (MAF = 5.4%; 11 heterozygotes vs. 90 homozygotes), rendering standard *r*^2^ and *D*′ metrics unreliable for LD block delineation. Moreover, this variant appeared as an isolated association peak, with no surrounding marker in linkage disequilibrium (maximum *r*^2^ = 0.10 within ±1 Mb) showing a concordant signal; the absence of LD support is inherent to such low-frequency variants and further limits confidence in this candidate. Although non-parametric evaluations confirmed the robust nature of the haplotype-phenotype association, this exploratory signal requires verification in larger validation populations. Second, cross-species inference via wheat transcriptomic data offers valuable but indirect insights; direct characterization of native *OatP5CDH* expression dynamics under LN stress represents an essential immediate objective. Third, the lack of genome-wide significant associations reflects statistical power limitations tied to our cohort size, which was reduced from the 186 accessions in the source study to the 103 accessions common to both datasets; expanded mapping panels are needed to fully resolve polygenic, small-effect networks. Furthermore, the phenotypes reported here are seedling-stage LN tolerance metrics expressed as LN/NN ratios; because the source study retained only these ratios rather than the absolute biomass values under each treatment, we could not distinguish whether the candidate allele promoted root growth under low nitrogen or reduced it under normal nitrogen, and these seedling-stage ratios should not be interpreted as direct evidence of improved whole-plant nitrogen-use efficiency or grain yield, which would require field-based evaluation across full growth stages. Finally, while our multi-omic approach successfully prioritized *P5CDH* on functional grounds, statistical association does not equal biological causation; future functional genomics efforts, including gene editing or overexpression assays, are required to validate the causal role of *P5CDH* in oat nutrient-stress adaptation.

## 5. Conclusions

In summary, this study provides a high-density, multi-model association analysis of low-nitrogen (LN) tolerance in hexaploid oat. No locus reached genome-wide significance, a pattern consistent with a complex, partly polygenic architecture that cannot be distinguished from the limited statistical power of the present panel. At a suggestive threshold, a candidate locus on chromosome 1D (1D:349,799,042) was associated with root dry weight, located 22.7 kb from *P5CDH*, a core proline-catabolism gene whose wheat ortholog is down-regulated under nitrogen deficiency. These findings nominate proline catabolism as a testable hypothesis for oat root plasticity under low nitrogen; while functional validation in larger panels is required to establish causality, this work provides a multi-omic framework and specific candidate genes to guide future genomic selection and validation studies.

## Figures and Tables

**Table 1:** Descriptive statistics and normality tests of the eleven LN tolerance traits (LN/NN ratios).

Trait	Mean	SD	CV (%)	SW (Raw)	SW (INT)
PH (plant height)	0.762	0.1	13.2	7.63 × 10^−3^	1
SPAD (chlorophyll content)	0.817	0.114	14	6.47 × 10^−7^	0.999
LL (leaf length)	0.824	0.088	10.7	4.44 × 10^−2^	0.998
LW (leaf width)	0.779	0.088	11.2	6.80 × 10^−1^	0.999
SFW (shoot fresh weight)	0.735	0.333	45.3	2.01 × 10^−8^	1
RFW (root fresh weight)	1.126	0.56	49.7	1.53 × 10^−11^	1
SDW (shoot dry weight)	0.94	0.612	65.1	4.30 × 10^−11^	1
RDW (root dry weight)	1.323	0.892	67.4	6.45 × 10^−10^	1
TRL (total root length)	1.345	0.397	29.5	8.49 × 10^−2^	1
RL (root length)	1.151	0.195	17	2.67 × 10^−1^	1
RA (root surface area)	1.263	0.352	27.9	1.59 × 10^−1^	0.999

Notes: LN/NN ratio, low-nitrogen value to normal-nitrogen value ratio; ratios < 1.0 and >1.0 indicate phenotypic reduction and increase under low-nitrogen stress, respectively. SD, standard deviation; CV, coefficient of variation. SW denotes the *p*-value derived from the Shapiro–Wilk normality test applied to the raw metric and the subsequent rank-based inverse normal transformation (rank-INT) values. All downstream analyses were performed on the normalized matrix.

**Table 2:** Summary of genome-wide association tests and Bayesian genetic architecture inference across eleven low-nitrogen tolerance traits.

Trait	MLM Bonf	MLM sugg	FarmCPU Bonf	FarmCPU sugg	PVE (95% CI)	rho (95% CI)	n_gamma
PH	0	83	0	99	0.134 [0.006, 0.441]	0.438 [0.017, 0.963]	76.2
SPAD	0	139	0	219	0.146 [0.005, 0.456]	0.471 [0.023, 0.971]	13.7
LL	0	72	0	83	0.136 [0.004, 0.457]	0.438 [0.015, 0.965]	25.3
LW	0	56	0	61	0.134 [0.003, 0.440]	0.502 [0.020, 0.975]	2.4
SFW	0	70	0	77	0.102 [0.003, 0.367]	0.443 [0.014, 0.972]	33.4
RFW	0	133	0	100	0.185 [0.009, 0.544]	0.492 [0.022, 0.971]	11.9
SDW	0	43	0	49	0.117 [0.004, 0.368]	0.448 [0.019, 0.971]	16
RDW	0	97	0	121	0.13 [0.006, 0.417]	0.445 [0.016, 0.961]	18.7
TRL	0	71	0	71	0.107 [0.002, 0.400]	0.468 [0.020, 0.968]	27.2
RL	0	288	0	315	0.229 [0.032, 0.529]	0.594 [0.048, 0.986]	5.4
RA	0	79	0	83	0.228 [0.028, 0.553]	0.542 [0.040, 0.981]	11.7

Notes: Bonf and sugg denote the number of SNPs exceeding the genome-wide Bonferroni-corrected threshold (*p* < 1.17 × 10^−9^) and the suggestive significance threshold (*p* < 1 × 10^−5^), respectively. PVE, *ρ*, and *n_γ_* represent the Bayesian sparse linear mixed model (BSLMM) posterior means for the total phenotypic variance explained (PVE; equivalent to the SNP-based narrow-sense heritability, *h*^2^), the proportion of PVE driven by sparse major-effect loci, and the estimated number of sparse-effect SNPs, respectively, calculated across the 5000 retained post-burn-in MCMC samples (50,000 sampling steps, recorded at a thinning interval of 10). PVE and ρ are reported as posterior means with 95% credible intervals in brackets; full SD and credible intervals for PVE, *ρ*, and n_gamma are provided in Table S7.

**Table 3:** Cross-trait association profile and allelic effect distribution for the prioritized candidate locus 1D:349,799,042.

Trait	MLM P	MLM β (SE)	FarmCPU P	FarmCPU Effect (SE)
RDW (root dry weight)	7.10 × 10^−7^	1.496 (0.282)	1.02 × 10^−6^	1.475 (0.283)
RFW (root fresh weight)	1.15 × 10^−3^	1.005 (0.300)	9.90 × 10^−4^	1.016 (0.299)
SDW (shoot dry weight)	3.99 × 10^−3^	0.899 (0.305)	4.80 × 10^−3^	0.880 (0.305)
TRL (total root length)	2.46 × 10^−2^	0.718 (0.314)	2.64 × 10^−2^	0.708 (0.314)
LW (leaf width)	9.03 × 10^−2^	−0.531 (0.310)	9.05 × 10^−2^	−0.529 (0.310)
RA (root surface area)	1.02 × 10^−1^	0.492 (0.298)	9.98 × 10^−2^	0.494 (0.298)
LL (leaf length)	1.92 × 10^−1^	0.413 (0.314)	2.22 × 10^−1^	0.386 (0.314)
SFW (shoot fresh weight)	2.70 × 10^−1^	0.352 (0.317)	2.83 × 10^−1^	0.342 (0.317)
PH (plant height)	4.67 × 10^−1^	0.231 (0.316)	5.41 × 10^−1^	0.194 (0.316)
SPAD (chlorophyll content)	6.85 × 10^−1^	0.129 (0.317)	7.75 × 10^−1^	0.091 (0.317)
RL (root length)	7.88 × 10^−1^	0.084 (0.312)	7.68 × 10^−1^	0.092 (0.311)

Notes: The target index variant is characterized as 1D:349,799,042:G:A (minor allele: A; minor allele frequency, MAF = 5.4%). *β* and effect denote the estimated additive genomic effects of the minor allele (A) derived from the MLM and FarmCPU algorithms, respectively, applied to rank-based inverse normal transformed (rank-INT) phenotypes; therefore, the estimates are in SD units, not raw biomass. SE, standard error; 95% CI = *β* ± 1.96 × SE. The proportion of phenotypic variance explained by the index variant (*R*^2^) was computed as 2*p*(1 − *p*)*β*^2^, where *p* is the minor allele frequency and *β* is the additive effect; this yielded *R*^2^ = 0.23 for RDW. Positive estimates indicate trait values are enhanced by the minor allele. Downstream verification metrics are cross-referenced to Figure 3 for the haplotype–phenotype segregation profile (two-sided Wilcoxon rank-sum test, *p* = 1.36 × 10^−5^; population-stratification adjustment via PC1, *p* = 0.619) and Table S4 for detailed functional annotations of the structurally mapped *P5CDH* gene model.

## Data Availability

The whole-genome resequencing genomic dataset analyzed during the current study was previously generated by our group and is publicly available in the NCBI Sequence Read Archive (SRA) under Bioproject accession number PRJNA921897 [27]. The phenotypic data were generated in the source study by Chai [28] (Master’s thesis, Inner Mongolia Agricultural University, 2024), and the per-accession values analyzed here are provided in Table S1. All analyses were performed with publicly available, versioned software, and the filtering parameters and association settings are specified in Section 2.2 and Section 2.5; the analysis scripts are provided as Supplementary Material (File S1).

## Supplementary Materials

The following supporting information can be downloaded at https://www.mdpi.com/article/10.3390/genes17101314/s1: Figure S1: Boxplots of the LN/NN ratios for the eleven traits; Figure S2: Manhattan plots of the MLM GWAS for the remaining ten traits; Figure S3: Quantile–quantile plots for the remaining ten traits; Table S1: Phenotypic data for 103 oat accessions across thirteen measured traits (eleven analyzed); Table S2: Full list of suggestive loci (MLM and FarmCPU intersection); Table S3: Functional annotations of candidate genes; Table S4: Candidate gene comparison between the prioritized 1D locus and the strongest RDW signal (3D);Table S5: Complete GO/KEGG enrichment results; Table S6: Cross-species comparison of the PEPC nitrogen-response genes; Table S7: BSLMM posterior means, SD, and 95% credible intervals for PVE, ρ, and n_γ across the eleven traits; Table S8: Per-trait candidate-gene counts and GO/KEGG enrichment results. File S1: Analysis scripts (Scripts collection, located in the “scripts” directory of the supplementary package).

## Abbreviations

The following abbreviations are used in this manuscript:

BSLMM	Bayesian sparse linear mixed model
CNKI	China National Knowledge Infrastructure
CV	coefficient of variation
FarmCPU	fixed and random model circulating probability unification
GO	gene ontology
GS	genomic selection
GWAS	genome-wide association study
HWE	Hardy–Weinberg equilibrium
INT	rank-based inverse normal transformation
KEGG	Kyoto Encyclopedia of Genes and Genomes
LD	linkage disequilibrium
LL	leaf length
LN	low nitrogen
LW	leaf width
MAF	minor allele frequency
MAS	marker-assisted selection
MCMC	Markov chain Monte Carlo
MLM	mixed linear model
NN	normal nitrogen
NUE	nitrogen use efficiency
*P5CDH*	Δ^1^-pyrroline-5-carboxylate dehydrogenase
PCA	principal component analysis
PEPC	phosphoenolpyruvate carboxylase
PH	plant height
PVE	phenotypic variance explained
QC	quality control
QTL	quantitative trait locus
RA	root surface area
RDW	root dry weight
RFW	root fresh weight
RL	root length
ROS	reactive oxygen species
SDW	shoot dry weight
SFW	shoot fresh weight
SNP	single-nucleotide polymorphism
SPAD	chlorophyll content
TDW	total dry weight
TFW	total fresh weight
TRL	total root length

## Appendix A. Plant Materials, Hydroponic Cultivation, and Phenotyping

### Appendix A.1. Seed Sterilization and Germination

For each oat accession, 70 plump, uniform seeds were selected and surface-sterilized with 75% (*v*/*v*) ethanol for 30 s, then rinsed three times with sterile distilled water. The seeds were soaked in the dark at 20 °C for 24 h until radicle emergence, then transferred to Petri dishes and germinated in darkness at 20 °C (day)/15 °C (night) with 75% relative humidity.

### Appendix A.2. Hydroponic Cultivation and Nitrogen Treatments

When approximately 80% of radicles had reached 1 cm, seedlings were transferred to 96-cell hydroponic seedling trays each containing 900 mL of complete Hoagland nutrient solution; the nutrient solution was renewed every 3 d. Seedlings were grown in a controlled-environment growth chamber at 20 °C (day)/16 °C (night) under a 16 h light/8 h dark photoperiod.

Two nitrogen treatments were imposed beginning at the three-leaf-one-heart stage (three fully expanded leaves with the fourth leaf just emerging): normal nitrogen (NN, 2 mmol/L NH_4_NO_3_) and low nitrogen (LN, 0.2 mmol/L NH_4_NO_3_). Each treatment was applied to three biological replicates. N-deficient Hoagland base solution (900 mL per tray) was used, with nitrogen supplied by adding liquid ammonium nitrate solution to reach the target NN or LN concentration. After 15 d of nitrogen treatment, the 13 low-nitrogen-tolerance traits were measured.

### Appendix A.3. Nutrient Solution Composition

Complete and nitrogen-free Hoagland base solutions were purchased from Beijing Coolaber Co., Ltd. (Beijing, China). The complete Hoagland solution contained (per liter): KNO_3_ 506 mg, NH_4_NO_3_ 80 mg, KH_2_PO_4_ 136 mg, MgSO_4_ 241 mg, FeNaEDTA 36.7 mg, KI 0.83 mg, H_3_BO_3_ 6.2 mg, MnSO_4_ 22.3 mg, ZnSO_4_ 8.6 mg, Na_2_MoO_4_ 0.25 mg, CuSO_4_ 0.025 mg, CoCl_2_ 0.025 mg, and Ca(NO_3_)_2_ 945 mg. In the nitrogen-free Hoagland solution, the three nitrogen-containing salts (KNO_3_, NH_4_NO_3_, Ca(NO_3_)_2_) were replaced by K_2_SO_4_ (435 mg/L) and MnSO_4_·H_2_O (16.9 mg/L), and the Ca^2+^ removed with Ca(NO_3_)_2_ was compensated by CaCl_2_ (444 mg/L); all other components were unchanged. Final nitrogen concentrations were 2 mmol/L (NN) and 0.2 mmol/L (LN), supplied solely as NH_4_NO_3_ (Appendix A.2); pH, aeration, and light intensity were not recorded in the source study.

### Appendix A.4. Phenotyping

All measurements were taken between 09:00 and 12:00. Thirteen traits were recorded as relative values (LN/NN ratio) to standardize for inherent growth differences among accessions:

- SPAD chlorophyll content (SPAD): measured on the selected plants using a handheld LYS-4N chlorophyll meter.
- Plant height (PH): distance from the root base to the tip of the highest leaf.
- Leaf length (LL) and leaf width (LW): the fully expanded leaf was measured for its maximum length (LL) and maximum width (LW).
- Shoot/root fresh weight (SFW, RFW, total fresh weight TFW): one plant per replicate was harvested and the shoot and root fresh weights were recorded immediately.
- Shoot/root dry weight (SDW, RDW, total dry weight TDW): the fresh samples were inactivated at 105 °C for 30 min and then dried at 80 °C to constant weight before weighing.
- Root architecture (total root length TRL, maximum root length RL, root surface area RA): roots were gently rinsed with a slow water stream, then scanned at 600 dpi on an Epson Expression 1000XL scanner (Epson, Suwa, Japan) using WinRHIZO (v2020a) for image acquisition; the images were subsequently analysed with RootNav v1.8.1 to obtain total root length, maximum root length, and root surface area. No formal image calibration or quality-control procedures were recorded in the source study.
